# Effect of neighborhood and individual-level socioeconomic factors on breast cancer screening adherence in a multi-ethnic study

**DOI:** 10.1186/s12889-023-17252-9

**Published:** 2024-01-02

**Authors:** Gillian Kasper, Mahsa Momen, Kristen A. Sorice, Kiara N. Mayhand, Elizabeth A. Handorf, Evelyn T. Gonzalez, Amie Devlin, Kirsten Brownstein, Nestor Esnaola, Susan G. Fisher, Shannon M. Lynch

**Affiliations:** 1 Temple University School of Medicine, Philadelphia, PA USA; 2 Johns Hopkins University Bloomberg School of Public Health, Baltimore, MD USA; 3 Fox Chase Cancer Center, 4th Floor Young Pavilion, 333 Cottman Avenue, Philadelphia, PA 19111 USA; 4 Houston Methodist, Houston, TX USA

**Keywords:** Breast cancer screening, Neighborhood, Health disparities, Social determinants of health

## Abstract

**Background:**

Although mammography can significantly reduce breast cancer mortality, many women do not receive their annual breast cancer screening. Differences in screening adherence exist by race/ethnicity, socioeconomic status (SES), and insurance status. However, more detailed investigations into the impact of neighborhood disadvantage and access to resources on screening adherence are lacking.

**Methods:**

We comprehensively examined the effect of individual social, economic, and demographic factors (*n* = 34 variables), as well as neighborhood level SES (nSES) indicators (*n* = 10 variables) on breast cancer screening adherence across a multi-ethnic population (*n* = 472). In this cross-sectional study, participants were surveyed from 2017 to 2018. The data was analyzed using univariate regression and LASSO for variable reduction. Significant predictors were carried forward into final multivariable mixed-effect logistic regression models where odds ratios (OR), 95% confidence intervals and *p*-values were reported.

**Results:**

Nineteen percent of participants were non-adherent to breast screening guidelines. Race/ethnicity was not associated with adherence; however, increasing age (OR = 0.97, 95%CI = 0.95–0.99, *p* = 0.01), renting a home (OR = 0.53, 95%CI = 0.30–0.94, *p* = 0.04), food insecurity (OR 0.46, 95%CI = 0.22–0.94, *p* = 0.01), and overcrowding (OR = 0.58, 95% CI = 0.32–0.94, *p* = 0.01) were significantly associated with lower breast cancer screening adherence.

**Conclusion:**

Socioeconomic indicators at the individual and neighborhood levels impact low breast cancer screening adherence and may help to inform future screening interventions.

**Supplementary Information:**

The online version contains supplementary material available at 10.1186/s12889-023-17252-9.

## Background

Breast cancer is one of the most commonly diagnosed cancers in women, accounting for 30% of all female cancer cases in the United States [1, 2]. Behind lung cancer, breast cancer is the second leading cause of cancer-related death in women [3]. From 2014–2018 in the United States, the female breast cancer incidence rate was 129.1 per 100,000 women per year and the death rate was 20.1 per 100,000 women per year [4]. Breast cancer incidence and mortality rates in females vary by race/ethnicity [3]. Data pooled from 2012–2016 showed that the female breast cancer incidence rate differed among Non-Hispanic Whites (NHW) (130.8 per 100,000), Non-Hispanic Blacks (NHB) (126.7 per 100,000), and Hispanics (93.7 per 100,000) [3]. While breast cancer incidence was higher in NHW, NHB have a 40% higher breast cancer mortality rate compared to NHW [3]. Based on data collected during 2013–2017, the mortality rates for NHW, NHB, and Hispanic females were 20.3, 28.4, and 14.0 per 100,000, respectively [3]. Trends in national data are also similar to those in the state of New Jersey, Pennsylvania, and the city of Philadelphia; the breast cancer mortality rates for New Jersey, Pennsylvania, and Philadelphia are 20.9, 21.1 and 24.8 per 100,000, respectively [5, 6]. Similarly, breast cancer mortality rates are higher among Black women than White women in Pennsylvania and Philadelphia [5].

Breast cancer can be detected early, and studies show that mammographic screening can significantly reduce breast cancer deaths by 20–40% [7]. Various techniques such as digital breast tomosynthesis, contrast-enhanced mammography, ultrasound supplemented with mammography, magnetic resonance imaging (MRI), and positron-emission mammography can be utilized to screen for breast cancer [7]. The American Cancer Society (ACS) recommends women aged 45–54 get screened every year, while women 55 and older can be screened every other year or yearly depending on their preference [8]. In addition, the ACS recommends that women aged 40–44 have the opportunity to receive a mammogram yearly [8]. Even though breast cancer screening significantly reduces mortality, less than half of all eligible women get a mammogram annually [9]. Black women are more likely than White women to be diagnosed at a later breast cancer stage because of lower breast cancer screening frequency, to have longer time in-between screenings, and to not have timely follow-up appointments after an abnormal screening result [10–13]. In comparison to Whites, Blacks and Hispanics experience longer times to diagnosis [13]. Hence, elimination of racial disparities in breast cancer screening practices is of critical public health importance to decrease breast cancer deaths in the U.S.

The National Institute on Minority Health and Health Disparities (NIMHD) created a multilevel, multidomain conceptual framework to help study racial disparities in cancer outcomes and to promote health equity [14]. This framework suggests biological, behavioral, physical and built environment, sociocultural environment, and health care system domains each can have influence on the individual, community, and society to impact cancer outcomes [14]. In the context of breast cancer screening, the NIMHD research framework suggests that a number of factors contribute to disparities including: socio-demographics, economics, perceived discrimination, cancer knowledge/beliefs, cancer-related risk behaviors, health literacy, social network support, and neighborhood-level variables, but studies that consider the impact of all these domains on an individual’s screening practices are limited. The NIMHD conceptual framework is further supported by other existing theories, including the fundamental cause theory, which also supports the consideration of these many domains, given these multiple factors (e.g. income and associated gain of power, social connections) collectively contribute to socioeconomic status (SES) and subsequent access to resources that can help or hinder health and health-related behaviors like cancer screening. Identifying these factors that contribute to resource inequities can inform future interventions to improve access to cancer screening [15].

The majority of prior breast cancer screening studies focus on racial/ethnic differences and/or socioeconomic status (SES), which can be defined in terms of a person’s own education, income, or employment [16, 17]. Previous studies have found women between the ages of 50 to 64 without a usual source of care, women living below the federal poverty level, and women who were uninsured or publicly insured were less likely to report a recent mammogram [18]. As a result, such women were more likely to have reduced breast cancer survival due to limited access to care [19–22]. Further, previous studies among Medicare patients have found that low-SES patients utilized less preventative care compared to higher SES patients even though they had insurance [16]. Additionally, food insecurity may contribute to poor breast cancer outcomes, particularly among low-income, ethnic minority, and female-headed households [23–26], but this has not been studied in the context of screening.

Beyond SES, additional studies have found that women who report perceived medical discrimination or medical mistrust [27, 28] were less likely to undergo breast cancer screening in comparison to women who did not report perceived discrimination or mistrust [29]. Women who have never been screened, or are overdue for screening, have less knowledge regarding screening guidelines, lack of social support, and lack of access to care, and for these reasons are not as likely to follow recommendations and utilize healthcare [27, 28].

In addition to the aforementioned individual-level factors, neighborhood disadvantage, leading to limited neighborhood access to health services have been shown to have an impact on breast cancer incidence, stage at diagnosis, and survival, but this has not been well-studied in regard to breast cancer screening adherence [26]. Studies suggest that people living in areas with higher levels of deprivation and rural areas have lower rates of cancer screening [30], and are more likely to be diagnosed at more advanced stages of breast cancer and have a poorer prognosis due to lack of access to quality care [31]. However, a limitation of these prior studies is that they did not include comprehensive assessments that investigated these associations in the context of other relevant individual-level factors from the NIMHD framework that could be associated with breast cancer screening adherence such as social/emotional support and quality of healthcare. Further, these studies have not assessed the impact of common neighborhood-level variables on breast cancer screening outcomes, alone and in the context of other relevant individual-level factors, related to socioeconomic status (neighborhood SES; nSES), such as deprivation indices [32], housing indicators (renting vs owning a home) or racial segregation [33]. According to fundamental cause theory, it is important to comprehensively assess various domains, including those impacting SES and access to resources associated with breast cancer screening in order to understand how to improve screening without further widening social inequalities in health [15]. This comprehensive approach will further help to avoid the development of interventions aimed at changing behaviors that are greatly influenced by factors outside a person’s behavior, including the environment in which they live. Therefore, under the guidance of the National Institute on Minority Health and Health Disparities (NIMHD) Research Framework [14], the goal of our study was first to comprehensively and systematically assess the impact of 10 neighborhood and 34 individual-level factors that impact breast cancer screening adherence in a multiethnic, underserved cohort from the Philadelphia, Pennsylvania and New Jersey areas utilizing high-dimenstional machine learning methods. Next, in support of principles from the fundamental cause theory, identification of variables associated with breast cancer screening adherence would then be used to provide insights into local interventions, including where and how to address disparities in breast cancer screening adherence in these underserved areas in ways that address the health burden, but do not increase social inequities.

## Methods

### Study population

Participants were identified from the Population Health Assessment (PHA) study. As part of the PHA, 1,000 individuals residing in underserved communities in the Fox Chase Cancer Center (FCCC) catchment area (including Philadelphia and southern suburban New Jersey counties) were administered surveys between July 2017 and May 2018. Two recruitment approaches were utilized: Half of the participants (*n* = 500) were recruited via convenience sampling by the FCCC Office of Community Outreach. The remaining 500 participants were recruited through *Temple Health: Block-By-Block* (THB^3^), a population-based cohort study focused on addressing community health concerns in the Temple University Hospital (TUH) primary service area [34]. Recruitment was supplemented via snowball sampling and sporadic convenience sampling at neighborhood venues and community events. Participants were initially screened for residence in ZIP codes with any of the following characteristics: 1) medically underserved areas (as defined by the Health Resources and Services Administration) [35]; 2) “low education” (i.e., < 79% of residents graduated from high school); and 3) lack of health insurance or subsidized health insurance coverage (i.e., Medicaid or state-subsidized plans for low-income adults). At least 75% of participants met at least one of the aforementioned criteria, in order to ensure a diverse cohort.

Study eligibility included being 18 years of age or older, able to understand English or Spanish, residing in the FCCC catchment area, and willing to provide a residential address. Participant address information was geocoded to the census tract level and assigned a Federal Information Processing Standard (FIPS) code to allow for linkage of participant data to the census-tract neighborhood and neighborhood-level variables. We limited our analysis to participants age 40 and over based upon breast cancer screening guidelines and because data collection procedures only asked participants aged 40 or older to respond to cancer screening questions. Participants who reported having breast cancer were also included. A total of 472 participants were included in the study. This cross-sectional study was approved by the institutional review board at FCCC (IRB#17–8005).

### Study outcome

The primary outcome in this analysis was breast cancer screening adherence status (adherent vs. non-adherent), defined based on guidelines from ACS [8], the National Comprehensive Cancer Network (NCCN) [36], and the United States Preventive Services Task Force (USPTF) [37] at the time of study enrollment. We coded adherence using survey responses regarding receipt of mammography. Participants aged 45–54 who were of average risk (no personal history of breast cancer) were considered adherent if they reported having had a mammography within the last year. Participants were additionally coded as adherent if they received a mammogram in the past year and either 1) were aged 40–45 and of average risk, or 2) had a history of breast cancer. Individuals not meeting these requirements were labeled non-adherent, with the exception of participants aged 40–45 of average risk who did not report having a mammogram in the last year, as the screening recommendations at the time began at age 45 (Appendix Table S1A-outcome definitions; https://github.com/gilliankasper7/Effect-of-Neighborhood-Individual-Level-Socioeconomic-Factors-on-Breast-Cancer-Screening-Adherence.git).

### Individual-level variables

Under the guidance of the multilevel National Institute on Minority Health and Health Disparities (NIMHD) Research Framework [14], the PHA survey collected data across multiple key constructs based on previous literature related to cancer health disparities and breast cancer screening adherence: socio-demographics [16, 17], economic measures [18] (e.g. income), healthcare access [19–22] (e.g. inability to attend an appointment because of cost), perceived discrimination [27–29], cancer knowledge/beliefs [27, 28], cancer related risk behaviors [38–40], health literacy [41, 42], and social network support [27, 28]. (See Table 1 and Appendix Table S1B; https://github.com/gilliankasper7/Effect-of-Neighborhood-Individual-Level-Socioeconomic-Factors-on-Breast-Cancer-Screening-Adherence.git).

**Table 1:** Significant baseline characteristics of the total study population based on univariate logistic regression models

Total Study Population (*n* = 472)				
	**Non-Adherent**	**Adherent**	**Total**	***P*-Value**
	**(*N* = 91)**	**(*N* = 381)**	**(*N* = 472)**	
***Socio-Demographics***				
**Age**				0.27
Mean (SD)	58.9 (11.0)	57.4 (11.1)	58.2 (10.9)	
Median [Min, Max]	54.0 [46.0, 94.0]	57.0 [39.0, 86.0]	55.3 [39.0, 94.0]	
**Race/ethnicity**				0.02^*^
NHW	20 (22.0%)	98 (25.7%)	118 (25%)	
NHB	42 (46.2%)	222 (58.3%)	264 (55.9%)	
Hispanic	29 (31.9%)	60 (15.7%)	89 (18.9%)	
Missing	0 (0%)	1 (0.3%)	1 (0.2%)	
**Center**				< 0.01*
FCCC	58 (63.7%)	185 (48.6%)	243 (51.5%)	
TUH	33 (36.3%)	196 (51.4%)	229 (48.5%)	
**Education**				0.38
More than high school	50 (54.9%)	224 (58.8%)	274 (58.1%)	
High school	20 (22.0%)	92 (24.1%)	112 (23.7%)	
Less than high school	21 (23.1%)	64 (16.8%)	85 (18.0%)	
Missing	0 (0%)	1 (0.3%)	1 (0.2%)	
**Marital status**				0.15
Married	23 (25.3%)	125 (32.8%)	148 (31.4%)	
Not Married	68 (74.7%)	253 (66.4%)	321 (68.0%)	
Missing	0 (0%)	3 (0.8%)	3 (0.6%)	
**Country of birth**				0.06
USA	79 (86.8%)	354 (92.9%)	433 (91.7%)	
Foreign	12 (13.2%)	27 (7.1%)	39 (8.3%)	
**Home rental or ownership**				0.01^*^
Rent/Other	54 (59.3%)	171 (44.9%)	225 (47.7%)	
Own	37 (40.7%)	210 (55.1%)	247 (52.3%)	
**Insurance type**				0.60
Government (Veteran)	3 (3.3%)	9 (2.4%)	12 (2.5%)	
Medicaid	33 (36.3%)	119 (31.2%)	152 (32.2%)	
Medicare	20 (22.0%)	101 (26.5%)	121 (25.6%)	
Not Insured	9 (9.9%)	27 (7.1%)	36 (7.6%)	
Private	25 (27.5%)	124 (32.5%)	149 (31.6%)	
Missing	1 (1.1%)	1 (0.3%)	2 (0.4%)	
***Economic Variables***				
**Household income**				.17^*^
Low (Less than $10,000 to under $20,000)	39 (42.9%)	141 (37.0%)	180 (38.1%)	
Mid ($20,000 to under $75,000)	35 (38.5%)	127 (33.3%)	162 (34.3%)	
High (above $75,000)	9 (9.9%)	74 (19.4%)	83 (17.6%)	
Not sure/Refused	8 (8.8%)	39 (10.2%)	47 (10.0%)	
**How often in the past 12 months would you say you were worried or stressed about having enough money to pay your rent/mortgage (financial insecurity)?**				0.24
Always/Usually	24 (26.4%)	83 (21.8%)	107 (22.7%)	
Sometimes	29 (31.9%)	95 (24.9%)	124 (26.3%)	
Never/Rarely	33 (36.3%)	183 (48.0%)	216 (45.8%)	
Not sure/Refused	5 (5.5%)	20 (5.2%)	25 (5.3%)	
**How often in the past 12 months would you say you were worried or stressed about having enough money to buy nutritious meals (food insecurity)?**				< 0.01^*^
Always/Usually	19 (20.9%)	58 (15.2%)	77 (16.3%)	
Sometimes	34 (37.4%)	85 (22.3%)	119 (25.2%)	
Never/Rarely	38 (41.8%)	236 (61.9%)	274 (58.1%)	
Missing	0 (0%)	2 (0.5%)	2 (0.4%)	
**Which one of these phrases comes closest to your own feelings about your household’s income these days?**				0.35
Living comfortably on present income	26 (28.6%)	113 (29.7%)	139 (29.4%)	
Getting by on present income	36 (39.6%)	176 (46.2%)	212 (44.9%)	
Finding it difficult/very difficult on present income	29 (31.9%)	86 (22.6%)	115 (24.4%)	
Missing	0 (0%)	6 (1.6%)	6 (1.3%)	
***Healthcare Access***				
**In the past 12 months was there a time when you needed to see a doctor but could not because of the cost?**				0.09
No	73 (80.2%)	331 (86.9%)	404 (85.6%)	
Yes	18 (19.8%)	49 (12.9%)	67 (14.2%)	
Missing	0 (0%)	1 (0.3%)	1 (0.2%)	
**About how long has it been since you last visited a doctor for a routine checkup?**				0.02
5 or more years ago	2 (2.2%)	0 (0%)	2 (0.4%)	
Within the past 5 years (more than 2 years ago but less than 5 years ago)	8 (8.8%)	9 (2.4%)	17 (3.6%)	
Within the past year/Within past 2 years	81 (89.0%)	371 (97.4%)	452 (95.8%)	
Missing	0 (0%)	1 (0.3%)	1 (0.2%)	
**Overall, how would you rate the quality of health care you received in the past 12 months?**				0.06^*^
Excellent/Very Good	46 (50.5%)	239 (62.7%)	285 (60.4%)	
Good	31 (34.1%)	108 (28.3%)	139 (29.4%)	
Fair/Poor	9 (9.9%)	28 (7.3%)	37 (7.8%)	
Not sure/Refused	5 (5.5%)	6 (1.6%)	11 (2.3%)	
***Perceived Discrimination***				
**Within the past 12 months, do you feel you were treated worse than, the same as, or better than people of other races?**				0.12
Better than other races	5 (5.5%)	43 (11.3%)	48 (10.2%)	
Only encountered people of the same race/The same as other races	67 (73.6%)	278 (73.0%)	345 (73.1%)	
Worse than other races	15 (16.5%)	41 (10.8%)	56 (11.9%)	
Missing	4 (4.4%)	19 (5.0%)	23 (4.9%)	
**Within the past 12 months, when seeking health care, do you feel your experiences were worse than, the same as or better than for people of other races?**				0.25
Better than other races	4 (4.4%)	40 (10.5%)	44 (9.3%)	
Only encountered people of the same race/The same as other races	69 (75.8%)	303 (79.5%)	372 (78.8%)	
Worse than other races	6 (6.6%)	20 (5.2%)	26 (5.5%)	
Not sure/Refused	6 (6.6%)	15 (3.9%)	21 (4.4%)	
Missing	6 (6.6%)	3 (0.8%)	9 (1.9%)	
***Cancer Knowledge/Beliefs***				
**When I think about cancer, I automatically think about death**				0.59
Strongly agree/Somewhat agree	39 (42.9%)	186 (48.8%)	225 (47.7%)	
Strongly disagree/Somewhat disagree	51 (56.0%)	191 (50.1%)	242 (51.3%)	
Not sure/Refused	1 (1.1%)	4 (1.0%)	5 (1.0%)	
**There’s not much you can do to lower your chances of getting cancer**				0.09
Strongly agree/Somewhat agree	40 (44.0%)	135 (35.4%)	175 (37.1%)	
Strongly disagree/Somewhat disagree	47 (51.6%)	239 (62.7%)	286 (60.6%)	
Not sure/Refused	4 (4.4%)	7 (1.8%)	11 (2.3%)	
**There are so many different recommendations about preventing cancer, it's hard to know which ones to follow**				0.27
Strongly agree/Somewhat agree	68 (74.7%)	292 (76.6%)	360 (76.3%)	
Somewhat disagree/Strongly disagree	19 (20.9%)	83 (21.8%)	102 (21.6%)	
Not sure/Refused	4 (4.4%)	6 (1.6%)	10 (2.1%)	
**Cancer is most often caused by a person's behavior or lifestyle**				0.06
Strongly agree/Somewhat agree	44 (48.4%)	235 (61.7%)	279 (59.1%)	
Somewhat disagree/Strongly disagree	44 (48.4%)	133 (34.9%)	177 (37.5%)	
Not sure/Refused	3 (3.3%)	13 (3.4%)	16 (3.4%)	
**Compared to other people your age, how likely do you think you are to get cancer in your lifetime?**				0.19
Very Likely/Likely	19 (20.9%)	113 (29.7%)	132 (28.0%)	
Neither unlikely nor likely	30 (33.0%)	92 (24.1%)	122 (25.8%)	
Very unlikely/Unlikely	20 (22.0%)	83 (21.8%)	103 (21.8%)	
Not sure/Refused	4 (4.4%)	25 (6.6%)	29 (6.1%)	
Missing	18 (19.8%)	68 (17.8%)	86 (18.2%)	
**I’d rather not know my chance of getting cancer**				0.28
Strongly agree/Somewhat agree	31 (34.1%)	103 (27.0%)	134 (28.4%)	
Strongly disagree/Somewhat disagree	40 (44.0%)	205 (53.8%)	245 (51.9%)	
Not sure/Refused	2 (2.2%)	5 (1.3%)	7 (1.5%)	
Missing	18 (19.8%)	68 (17.8%)	86 (18.2%)	
**How worried are you about getting cancer?**				0.07
Moderately/Extremely	8 (8.8%)	80 (21.0%)	88 (18.6%)	
Somewhat	18 (19.8%)	57 (15.0%)	75 (15.9%)	
Not at all/Slightly	47 (51.6%)	173 (45.4%)	220 (46.6%)	
Not sure/Refused	0 (0%)	3 (0.8%)	3 (0.6%)	
Missing	18 (19.8%)	68 (17.8%)	86 (18.2%)	
**At what age do you think women are supposed to start having mammograms?**				0.34
Correct	7 (7.7%)	15 (3.9%)	22 (4.6%)	
Incorrect	84 (92.3%)	360 (94.5%)	444 (94.1%)	
Not sure/Refused	0 (0%)	6 (1.6%)	6 (1.3%)	
***Cancer-Related Risk Behaviors***				
**Current smoker**				0.86
No	73 (80.2%)	305 (80.1%)	378 (80.1%)	
Yes	17 (18.7%)	75 (19.7%)	92 (19.5%)	
Missing	1 (1.1%)	1 (0.3%)	2 (0.4%)	
**Alcohol use**				< 0.01^*^
No	60 (65.9%)	195 (51.2%)	255 (54.0%)	
Yes	30 (33.0%)	185 (48.6%)	215 (45.6%)	
Missing	1 (1.1%)	1 (0.3%)	2 (0.4%)	
**Diabetes**				0.75
No	66 (72.5%)	281 (73.8%)	347 (73.5%)	
Yes	25 (27.5%)	98 (25.7%)	123 (26.1%)	
Missing	0 (0%)	2 (0.5%)	2 (0.4%)	
**Body mass index (BMI)**				0.20
Mean (SD)	30.1 (7.15)	31.2 (8.00)	30.7 (7.34)	
Median [Min, Max]	28.0 [20.1, 52.9]	29.3 [17.3, 74.0]	29.0 [17.0–74.0]	
Missing	1 (1.1%)	7 (1.8%)	8 (1.5%)	
***Health Literacy***				
**How difficult is it for you to understand information that doctors, nurses etc., tell you?**				0.10
Very Difficult/Somewhat difficult	22 (24.2%)	63 (16.5%)	85 (18.0%)	
Very easy/Somewhat easy	69 (75.8%)	316 (82.9%)	385 (81.6%)	
Missing	0 (0%)	2 (0.5%)	2 (0.4%)	
***Social Network Support***				
**How often do you get the social and emotional support you need?**				0.10^*^
Always/Usually	56 (61.5%)	246 (64.6%)	302 (64.0%)	
Rarely/Never	8 (8.8%)	50 (13.1%)	58 (12.3%)	
Sometimes	27 (29.7%)	83 (21.8%)	110 (23.3%)	
Missing	0 (0%)	2 (0.5%)	2 (0.4%)	
***Neighborhood-level variables***				
**Proportion living in same house as 1 year ago**				0.21
Mean (SD)	0.871 (0.0694)	0.881 (0.0642)	0.877 (0.0660)	
Median [Min, Max]	0.888 [0.677, 0.982]	0.892 [0.549, 0.988]	0.887 [0.55–0.988]	
**Proportion with poor English**				0.04
Mean (SD)	0.0904 (0.101)	0.0694 (0.0842)	0.0708 (0.0878)	
Median [Min, Max]	0.0466 [0.00, 0.375]	0.0353 [0.00, 0.422]	0.0354 [0, 0.422]	
**Proportion living in household isolation**				0.11
Mean (SD)	0.0645 (0.0844)	0.0506 (0.0708)	0.0499 (0.0744)	
Median [Min, Max]	0.0309 [0.00, 0.393]	0.0225 [0.00, 0.393]	0.0188[0, 0.393]	
**ICE-Income (Quartiles)**				0.86
1 Concentrated Poverty	58 (63.7%)	235 (61.7%)	293 (62.1%)	
2	15 (16.5%)	36 (9.4%)	51 (10.8%)	
3	9 (9.9%)	45 (11.8%)	54 (11.4%)	
4 Concentrated Affluence	9 (9.9%)	65 (17.1%)	74 (15.7%)	
**ICE-Race (Quartiles)**				0.02^*^
1 High Concentration of NHB	67 (73.6%)	267 (70.1%)	334 (70.8%)	
2	11 (12.1%)	50 (13.1%)	61 (12.9%)	
3	10 (11.0%)	44 (11.5%)	54 (11.4%)	
4 High Concentration of NHW	3 (3.3%)	20 (5.2%)	23 (4.9%)	
**ICE Race + Income (Quartiles)**				0.05^*^
1 Concentrated Poverty of NHB	68 (74.7%)	264 (69.3%)	332 (70.3%)	
2	6 (6.6%)	36 (9.4%)	42 (8.9%)	
3	11 (12.1%)	24 (6.3%)	35 (7.4%)	
4 Concentrated Affluence of NHW	6 (6.6%)	57 (15.0%)	63 (13.3%)	
**Yost Index (Quintiles)**				0.07
1 Low SES	50 (54.9%)	200 (52.5%)	250 (53.0%)	
2	18 (19.8%)	55 (14.4%)	73 (15.5%)	
3	11 (12.1%)	25 (6.6%)	36 (7.6%)	
4	4 (4.4%)	39 (10.2%)	43 (9.1%)	
5 High SES	7 (7.7%)	53 (13.9%)	60 (12.7%)	
Missing	1 (1.1%)	9 (2.4%)	10 (2.1%)	
**Median household income**				0.55
Mean (SD)	41400 (25700)	45900 (32600)	43650 (29150)	
Median [Min, Max]	32300 [14000, 136000]	32100 [11400, 187000]	32115 [11400, 187000]	
**% Overcrowding**				< 0.01^*^
% Overcrowding—High	58 (63.7%)	180 (47.2%)	238 (50.4%)	
% Overcrowding—Low	33 (36.3%)	201 (52.8%)	234 (49.6%)	
**% with access to transportation (1 or more vehicles)**				0.60
Mean (SD)	0.705 (0.193)	0.692 (0.208)	0.6985 (0.201)	
Median [Min, Max]	0.675 [0.343, 1.00]	0.658 [0.304, 1.00]	0.66 [0.304–1.00]	

^*^Denotes variables that are significant in the univariate analysis at *p* < 0.2 and identified as a predictor by LASSO (Appendix Table S1D; https://github.com/gilliankasper7/Effect-of-Neighborhood-Individual-Level-Socioeconomic-Factors-on-Breast-Cancer-Screening-Adherence.git)

### Neighborhood-level variables

Neighborhood-level measures found to be associated with cancer mortality or screening adherence [26, 30–33] in prior studies were included: 1) Neighborhood Stability; 2) Language Skills; 3) Household Isolation; 4) Household Income; 5) Crowding; 6) Transportation; 7) multiple Index of Concentration at the Extremes (ICE) measures [43–46]; 8) Yost Index [32, 47] (See Table 1 and Appendix Table S1B; https://github.com/gilliankasper7/Effect-of-Neighborhood-Individual-Level-Socioeconomic-Factors-on-Breast-Cancer-Screening-Adherence.git). Neighborhood data were obtained from the United States Census (Year 2010) and American Community Survey (2014–2018) at the census tract level. Neighborhood-level variables were linked to participant data using a Federal Information Processing Standard (FIPS) code at the census tract level in R (R Core Team, Vienna, Austria) [48].

### Statistical analysis

Continuous variables were summarized by means and medians, and categorical variables were summarized as percentages. Next, we ran univariate logistic regression models [49] to identify individual-level and neighborhood-level variables significantly associated with breast cancer screening adherence at a Wald *p*-value < 0.20 in the total study population (univariate p-values reported in Table 1) and individually for NHB, NHW, and Hispanic participants (univariate p-values and statistical equations are reported in Appendix Table S1C; https://github.com/gilliankasper7/Effect-of-Neighborhood-Individual-Level-Socioeconomic-Factors-on-Breast-Cancer-Screening-Adherence.git). This is similar to the high-dimensional computing approach our team previously developed, known as a neighborhood-wide association study (NWAS) [50]. As an additional variable reduction step and to account for the high degree of correlation within and across individual-level domains and neighborhood variables, we applied LASSO machine learning approaches [51] (Appendix Table S1D with statistical equation presented; https://github.com/gilliankasper7/Effect-of-Neighborhood-Individual-Level-Socioeconomic-Factors-on-Breast-Cancer-Screening-Adherence.git). Variables that were identified as predictors in LASSO (non-zero values) AND that were significant in the univariate analysis (*p*-value < 0.20) were marked with an asterisk (*) in Table 1 and were carried forward to mixed effect multivariable logistic regression models which accounted for neighborhood clustering effects [49, 51–53] (Appendix Table S1E with statistical equation presented; https://github.com/gilliankasper7/Effect-of-Neighborhood-Individual-Level-Socioeconomic-Factors-on-Breast-Cancer-Screening-Adherence.git). Next, we refined the multivariate model via backward elimination. A score test was run after each backward selection step until a *p* < 0.05 was estimated to determine the best model fit and the final model [53]. Odds ratios representing adherence, 95% confidence intervals (CI), and p-values from the final model in the total study population are reported in Table 2. Given small sample sizes, we were unable to stratify multivariable models by racial group, but univariate findings are presented in Appendix Table S1C (https://github.com/gilliankasper7/Effect-of-Neighborhood-Individual-Level-Socioeconomic-Factors-on-Breast-Cancer-Screening-Adherence.git). Thus, multivariable models using the total study population serve as the main findings.

**Table 2:** Final multivariable analysis: total population (*n* = 451)

	**Odds Ratio Estimate**	**Lower CI**	**Upper CI**	*p*-value^*^	Overall *P*- value^**^
***Socio-Demographics***					
**Age**	0.97	0.95	0.99	0.013	**0.01**
**Race/ethnicity**					**0.56**
NHB	1.20	0.50	2.89	0.682	
Hispanic	0.69	0.26	1.79	0.441	
NHW	ref				
**Center**					**0.01**
TUH	2.66	1.47	4.81	.0012	
FCCC	ref				
**Home rental or ownership**					**0.04**
Rent/Other	0.53	0.30	0.94	0.030	
Own	ref				
***Economic Variables***					
**Household income**					**0.12**
Low (Less than $10,000 to under $20,000)	0.50	0.17	1.46	0.202	
Mid ($20,000 to under $75,000)	0.36	0.13	0.98	0.045	
High (above $75,000)	ref				
Not sure/Refused	0.76	0.19	3.06	0.704	
**How often in the past 12 months would you say you were worried or stressed about having enough money to buy nutritious meals (food insecurity)?**					**0.01**
Always/Usually	0.46	0.22	0.94	0.033	
Sometimes	0.44	0.24	0.80	0.007	
Never/Rarely	ref				
***Neighborhood-level variables***					
**ICE Race + Income (Quartiles)**					**0.04**
1 Concentrated Poverty of NHB	0.45	0.13	1.55	0.203	
2	0.97	0.25	3.81	0.961	
3	0.16	0.04	0.73	0.018	
4 Concentrated Affluence of NHW	ref				
**% Overcrowding**					**0.01**
High	0.58	0.32	0.94	0.045	
Low	ref				

^*^p-value reported from Wald Test; ^**^p-value reported from score test

## Results

### Univariate analysis

All participants in the study (*n* = 472) were females between the ages of 39 and 94 (mean age 58.2 (SD 10.9)). Fifty-six percent of participants were NHB, 25% were NHW, and 19% self-identified as Hispanic. Nineteen percent of the total study population were considered to be non-adherent to breast cancer screening guidelines. Within the domains of socio-demographics, economic, healthcare access, cancer-related beliefs, social support, and neighborhood, there was general agreement between variables found to be significant in univariate analysis (*p* < 0.05) and LASSO analysis (see * variables in Table 1). This finding suggests our less stringent inclusion criteria to identify overlap between LASSO and univariate analysis at an initial *p* < 0.20 likely did not result in missing associations. We summarize the findings from these variables below.

Non-adherence significantly differed by race (*p* = 0.02) and healthcare center (*p* < 0.01). Compared to adherent study participants, non-adherence was more prevalent in females who rented their home (59.3% vs 44.9%, *p* = 0.01); were of low-income (42.9% vs 37%; *p* = 0.17); reported being usually/always worried about being able to afford nutritious meals (20.9% vs 15.2%; *p* < 0.01); rated the quality of health care received in the past year as fair/poor (9.9% vs 7.3%; *p* = 0.06); consumed alcohol in the past 30 days (33.0% vs 48.6%; *p* < 0.01); described that their social and emotional support needs were sometimes and rarely/never met (38.5% vs 34.9%; *p* = 0.1). Interestingly, across both adherent and non-adherent populations, over 90% of participants did not correctly identify the recommended age of 45 to start having a mammogram.

Regarding neighborhood-level variables, non-adherent study participants were more likely to live in neighborhoods with a high concentration of NHBs (73.6% vs 70.1%; *p* = 0.02); a high concentration of NHBs living in poverty compared to affluent White residents (74.7% vs 69.3%; *p* = 0.05), and in neighborhoods with high levels of overcrowding (63.7% vs 47.2%; *p* < 0.01). Neighborhood and individual-level variables significant in both univariate and LASSO were then further statistically analyzed in multivariable regression (Table 2).

### Multivariable analysis

Significant variables in the univariate analysis and LASSO were added to a full multivariable model (Appendix Table S1E- first model; https://github.com/gilliankasper7/Effect-of-Neighborhood-Individual-Level-Socioeconomic-Factors-on-Breast-Cancer-Screening-Adherence.git), and backward regression and score test comparisons were used to identify the final, best-fitting model (Table 2- final model). Variables described here are those that remained significant in the final model for the total population. Increasing age (one year increases) was shown to be associated with being non-adherent to breast cancer screening (OR = 0.97, 95% CI 0.95–0.99, *p* = 0.01). There were no significant differences in adherence by race; however, participants from TUH had significantly higher odds of breast cancer screening adherence than those seen at FCCC (OR = 2.66, 95% CI 1.47–4.81, *p* = 0.006) and 69% of the NHB population in this study came from TUH (Appendix Table S1C; https://github.com/gilliankasper7/Effect-of-Neighborhood-Individual-Level-Socioeconomic-Factors-on-Breast-Cancer-Screening-Adherence.git). Participants who stated that they rented rather than owned a home had significantly lower odds of breast cancer screening adherence (OR = 0.53, 95% CI 0.30–0.94, *p* = 0.04). Women who reported that in the past 12 months they were always/usually stressed (OR = 0.46, 95% CI 0.22–0.94, *p* = 0.01) and sometimes stressed (OR = 0.44, 95% CI 0.24–0.80, *p* = 0.01) about having enough money to buy nutritious meals showed lower odds of breast cancer screening adherence than those who never/rarely experienced food insecurity. Overall, ICE Race/Income was significantly associated with decreased odds of breast cancer screening adherence (*p* = 0.04). Lastly, participants who lived in areas with a high percentage of overcrowding (OR = 0.58, 95% CI 0.32–0.94, *p* = 0.01) had lower odds of screening adherence for breast cancer than those participants living in areas with low overcrowding conditions.

## Discussion

Using the NIMHD’s multi-level conceptual framework, we are one of the first studies to apply high-dimensional computing methods to comprehensively assess and identify individual and neighborhood-level variables that may impact breast cancer screening adherence within a diverse and under-served study population from Philadelphia and New Jersey. These statistical approaches allowed us to account for correlation and consider contextual effects of highly related individual and neighborhood variables to comprehensively assess and pinpoint which variables are most contributing to breast cancer screening adherence in our study population. Results are in line with the fundamental cause theory, finding that specific socioeconomic factors and access to resources likely play a role in adherence [15]. These findings will be used to develop future interventions that not only address screening behavior, but that also address or target populations with limited resources and social indicators that impede adherence to breast cancer screening in our underserved population [15].

In comparison to the national average (66.7% of females over the age of 40), 80.7% of our study population was adherent to breast cancer screening recommendations [54]. This relatively high rate of breast cancer screening adherence in our study population is supported by prior population-based studies conducted in Philadelphia showing 84% of age eligible women reporting a mammography in the last year [55]. Further, results in our study demonstrating that NHB women have higher breast cancer screening adherence compared to NHW and Hispanic women in univariate analysis are also supported by prior literature at the national and local Philadelphia levels [53, 54]. In multivariate analysis, racial differences in adherence were not significant but could be attributable to the race/ethnic profile of our underserved population, which consisted primarily of Black participants (55.9%).

In comparison to the FCCC testing site, TUH was associated with increased adherence. This could reflect differences in sampling approaches (TUH was population-based and supplemented by snowball and sporadic convenience sample; FCCC was recruited primarily through convenience sampling). A number of screening and community interventions targeting the Black community in the TUH catchment area could also have contributed to higher breast cancer screening adherence rates. For example, FCCC has a mammography mobile screening van that primarily serves the TUH community and accounted for approximately 25% of screen-detected breast cancers diagnosed at the combined FCCC/TUH medical campuses. Increased age was associated with non-adherence. This may be because some women aged 55 and older opted to get screened every other year, rather than yearly, based on the recommendations [8]. However, across adherent and non-adherent groups, women generally did not appear to know what age to start getting a mammography. This suggests that educational interventions are needed, as well as more detailed insights into the frequency of mammography in the 55 and older groups at average risk of breast cancer.

Despite relatively high breast cancer screening rates among our study population, we’ve identified key structural and socioeconomic factors that may play a significant role in screening non-adherence. Our study found associations between breast cancer screening nonadherence and food insecurity, renting a home, and overcrowding. Food insecurity is a critical social determinant of health that is associated with low income, is often under-recognized [56], and to our knowledge, has not been previously studied in the context of breast cancer screening adherence. Thus, we are first to report this association with screening adherence. Previous studies have shown that low-income families may postpone their medical needs or underuse healthcare services in order to prioritize food spending due to budgetary limitations [56–58]. Not only are individuals who experience food insecurity more likely to consume nutrient-poor diets contributing to risk factors of breast cancer, but they are also likely to experience depression, anxiety, and psychological distress [26]. Adherence to cancer screenings in general is likely and understandably not a leading priority for individuals experiencing food insecurity.

Furthermore, renting versus owning a home was examined as a marker of financial insecurity. Renting a home has been previously studied in regards to cancer incidence [50], and associations have been identified in a similar study with non-adherence to colorectal cancer screenings [59]. Homeownership is a socioeconomic indicator that can be used not only to further assess financial stability but also as an economic marker to evaluate access to healthcare services. A previous study found that homeowners have significantly better quality of housing, wealth, physical, and mental health, whereas renting a home is often associated with lower income levels, food insecurity, overcrowding, and poorer health outcomes [60]. This relationship is exacerbated when homeownership is stratified by race/ethnic group [60].

Overcrowding was also significantly associated with breast cancer screening non-adherence. Prior literature supporting or rejecting this association has varied. Using 2010 census tract data, a previous study based in Texas examined mammography screening adherence and found that patients who lived in a crowded environment were less likely to have up-to-date mammography screening [61]. Although unmeasured, Calo and colleagues attribute the identified interaction effect of overcrowding and age in their breast cancer screening adherence study to the influence of social network norms and beliefs [61]. We, however, found none of our cancer knowledge/beliefs or social support variables to be significant in our final model. Dailey and colleagues examined neighborhood-level crowding in Connecticut using 1990 census tract data and found it to be associated with breast cancer screening nonadherence in White women but non-significant in Black women [62]. Our sample size was not large enough to assess potential differences in adherence stratified by race/ethnic group. It is important to note that prior literature assessing the relationships between overcrowding and breast cancer screening adherence referenced here did so in different geographical areas of the US where crowding rates have changed and are different than the geographical area of our study. Based on 2000 housing census data on crowding, Pennsylvania and New Jersey were marked at 1.9% crowded and 5.0% crowded respectively [63]. This is versus 9.4% crowded for Texas and 2.8% crowded for Connecticut where these other studies mentioned were done [63].

In the multivariable analysis, ICE Race/Income showed significance with concentrated deprivation of NHB demonstrating lower breast cancer screening adherence versus concentrated privilege of NHW. Similarly, a previous study also found that for NHB women, neighborhood-level median household income and neighborhood-level poverty were significantly associated with mammography nonadherence, which may be due to limited access to care and resources supporting good health and preventative services [62]. In a study examining spatial disparities in breast cancer survival in New Jersey, Wiese and colleagues found that ICE Income was associated with breast cancer survival [64], however, prior research examining the association between ICE measures and breast cancer screening is lacking.

Results of our study demonstrate the critical role that conjoined environmental and socioeconomic factors play in an individual’s ability to prioritize screening adherence and other preventative services. Thus, these results support not only the NIMHD’s theoretical framework that both neighborhood and individual factors play a role, but also the fundamental cause theory, given translation of these findings into future, local interventions would likely need to consider the context of SES factors, including food insecurity, housing, if behavior change related to breast cancer screening is desired. More specifically, an assumption of the fundamental cause theory is that individuals are able to utilize resources to improve health [15, 65]; however, when given the ability or choice to participate in cancer screening, it is possible that persons from underserved backgrounds may not be able to participate due to competing factors related to social and economic circumstances resulting from influences at multiple levels- family, social support, society and policy [65]. Thus, future studies and interventions related to cancer screening should both address and continue to evaluate the effect new interventions have not only on health outcomes, but the socioeconomic circumstances associated with person, neighborhood, and social policies believed to also impact health outcomes. This is important to ensure that the intervention is not reproducing inequities by only benefiting individuals from a higher socioeconomic background who may have more access to knowledge and newly available resources [15]. In future interventions, one way we could address this in our study population is the utilization of a mobile mammography screening unit that brings breast cancer screening to low socioeconomic, underserved communities and housing developments, while also partnering with food bank services to address food insecurity. Over time, we would plan to continuously evaluate the impact of this dual intervention approach on screening adherence by socioeconomic circumstances through quantitative and qualitative assessments to ensure this intervention approach is not perpetuating or reproducing socioeconomic differences in adherence to screening.

This study has limitations to address. Since our study was cross-sectional and collected survey data at one point in time from a non-random study sample, it limited our ability to evaluate causal relationships. Using a nonprobability sampling approach to target participants in an underserved population may have introduced selection bias into our study. There is the possibility that the sample may not be an accurate representation of population-based socioeconomic or race/ethnic groups. Though our study utilized a multilevel framework to examine many variables believed to be associated with cancer disparities, our study was limited to measures that were chosen as part of the funded PHA supplement. It is possible that there are other variables that are associated with cancer screening that were not evaluated in this study. Moreover, our study relied on self-reported data regarding breast cancer screening adherence from patients, which may be influenced by recall bias and may not be as accurate as medical records. Furthermore, some questions in the PHA survey, such as cancer beliefs and knowledge, were only asked from participants who did not have a previous history of cancer, which resulted in missing data. As a result, these variables were not included in the multivariate models, however, it was included in the univariate results for transparency and in the hopes that the crude estimates may aid future studies. Furthermore, it is possible that some findings may be due to chance or that we may have missed some associations given our multistep statistical approach. This study analyzed variables in the context of existing social theories and frameworks that suggest the inter-relatedness of social factors such as income, education, effects on employment and ability to carry insurance as a result of employment, but here, we relied heavily on high dimensional computing approaches to reduce the number of variables to pinpoint which factors, in the context of one another, are most associated with our outcome to inform targeted interventions. In particular, the multivariable LASSO statistical approach was used to minimize the possibility of confounding factors and address any multicollinearity. However, it is still possible that confounding, interaction, and mediation effects exist regarding race, economic, and sociodemographic variables. This was an association study, but future causal inference studies in this domain could be warranted. While our multistep approach has been applied in other study settings and can be applied in other studies [59], it is likely that findings may differ and have limited generalizability in more rural, non-urban settings, given the sociodemographics and geographic location of the study population often vary across studies. Future studies with a larger, multiethnic cohort may better account for and minimize the impact of confounding factors and allow for stratified analyses by race/ethnicity and geographic location.

## Conclusion

After a systematic and methodologic assessment utilizing novel statistical approaches, this study identified both existing and new variables associated with breast cancer screening adherence, including age, center, homeownership, food insecurity, overcrowding, and ICE Race/Income. These results may help to identify individuals and communities who may benefit from targeted interventions regarding breast cancer screening and as such, could reduce disparities in breast cancer mortality by including intervention components that also address socioeconomic factors that affect screening uptake and health more broadly. These results also add to health disparities research in general in that we identified key social determinants of health that can either be addressed or mitigated in a future intervention or used to identify populations to target for breast cancer screening. In the future, surveillance data of the aforementioned variables could be used to guide breast cancer screening more effectively in our surrounding areas, and can help to ensure that interventions related to cancer screening are benefiting residents across the socioeconomic continuum. For example, screening efforts to identify patients who rent a home and who live in overcrowded conditions may be more efficient than targeting a specific race/ethnic group. Future investigations may benefit from assessing the role of race/ethnic and socioeconomic experiences on cancer screening adherence and outcomes in larger, multiethnic, socioeconomically diverse samples. This study demonstrates that comprehensive assessments in health disparities research at both the individual and neighborhood levels can help us to expand our understanding of the impact of health inequities on health outcomes, as well as potentially help to guide where and whom to target for interventions to promote cancer screening.

### Supplementary Information

**Additional file 1: Table S1A.** Outcome definitions. **Table S1B.** Description of individual and neighborhood-level variables. **Table S1C.** Significant baseline characteristics by race/ethnicity based on univariate regression models**. **Table S1D.** LASSO Model* Results: A cross validation method was used to estimate lambda, the tuning parameter. Based on the results, we chose the lambda value with the lowest cross validation error value to assess results. **Table S1E.** First multivariable analysis model* Results: Total Population (*n*=447).

## Data Availability

The datasets generated during and/or analyzed during the current study are not publicly available due to the consent provided by participants on the use of confidential data, but are available from the corresponding author on reasonable request.

## Abbreviations

ACS: American Cancer Society
CI: Confidence Interval
FCCC: Fox Chase Cancer Center
ICE: Index of Concentration at the Extremes
IRB: Institutional Review Board
FIPS: Federal Information Processing Standard
NCCN: National Comprehensive Cancer Network
nSES: Neighborhood Socioeconomic Status
OR: Odds Ratio
MRI: Magnetic resonance imaging
NHB: Non-hispanic Black
NHW: Non-hispanic White
NIMHD: National Institute on Minority Health and Health Disparities
PHA: Population Health Assessment
SES: Socioeconomic status
THB3: Temple Health Block by Block
TUH: Temple University Hospital
